# The Physics and Metaphysics of Social Powers: Bridging Cognitive Processing and Social Dynamics, a New Perspective on Power Through Active Inference

**DOI:** 10.3390/e27050522

**Published:** 2025-05-14

**Authors:** Mahault Albarracin, Sonia de Jager, David Hyland

**Affiliations:** 1 VERSES.ai, Los Angeles, CA 90016, USA; 2 Département d’informatique, Université du Québec à Montréal, Montréal, QC H3C 3P8, Canada; 3 Institut de Sante et Societe, Université du Québec à Montréal, Montréal, QC H3C 3P8, Canada; 4 Erasmus School of Philosophy, Erasmus University, 3062 PA Rotterdam, The Netherlands; dejager@esphil.eur.nl; 5 Noise Research Union (NRU); 6 Department of Computer Science, University of Oxford, Oxford OX1 2JD, UK; david.hyland@stx.ox.ac.uk

**Keywords:** power, scripts, active inference, narratives, semantic attraction, (shared) attention

## Abstract

Power operates across multiple scales, from physical action to complex social dynamics, and is constrained by fundamental principles. In the social realm, power is shaped by interactions and cognitive capacity: socially-facilitated empowerment enhances an agent’s information-processing ability, either by delegating tasks or leveraging collective resources. This computational advantage expands access to policies and buffers against vulnerabilities, amplifying an individual’s or group’s influence. In AIF, social power emerges from the capacity to attract attention and process information effectively. Our semantic habitat—narratives, ideologies, representations, etc.—functions through attentional scripts that coordinate social behavior. Shared scripts shape power dynamics by structuring collective attention. Speculative scripts serve as cognitive tools for low-risk learning, allowing agents to explore counterfactuals and refine predictive models. However, dominant scripts can reinforce misinformation, echo chambers, and power imbalances by directing collective attention toward self-reinforcing policies. We argue that power through scripts stems not only from associations with influential agents but also from the ability to efficiently process information, creating a feedback loop of increasing influence. This reframes power beyond traditional material and cultural dimensions, towards an informational and computational paradigm—what we term possibilistic power, i.e., the capacity to explore and shape future trajectories. Understanding these mechanisms has critical implications for political organization and technological foresight.

## 1. Introduction: The Nature of Power

The concept of *power* has a long history and can be explored at several scales. In this article, our interest is to explore the concept as a phenomenon that can be understood as unfolding across scales, by framing and formalizing it through active inference (AIF). In the context of social organization dynamics, one of the most compelling elucidations of what constitutes the nature of power was presented by Michel Foucault:

“...power must be understood in the first instance as the *multiplicity of force relations immanent in the sphere in which they operate and which constitute their own organization*; as the process which, through ceaseless struggles and confrontations, transforms, strengthens, or reverses them; as the support which these force relations find in one another, thus *forming a chain or a system*, or on the contrary, the *disjunctions and contradictions which isolate them from one another*; and lastly, as the *strategies* in which they take effect...”.

([1], pp. 92–93, our emphasis in italics)

Thus, the phenomenon of social power acquisition and maintenance is a complex, multidirectional process that resists simplistic or hierarchical schematization. Instead, it must be analyzed in terms of agent interactions within constraint regimes. Traditionally, socially-facilitated empowerment—such as that of the state or large corporations—has been framed as the execution of top-down strategies, tactics, and narratives (*scripts*, as argued later) that grant agents greater *leverage*, i.e., influence over future states. (We use “state” in the sense of “condition”, though the political connotation is relevant).

We recast this in terms of information-processing capacity: power emerges either through reliance on others to enact desired policies (*exploitation*, in biological and sociological senses) or through enhanced computational possibilities via task delegation (*exploration*). Social empowerment thus entails an increased ability to compute information, expanding a given state space’s evolution. Applying AIF, we analyze power as “the probability that one actor within a social relationship will be in a position to carry out [their] will despite resistance” ([2], p. 152) in terms of *actual* probabilities. (We challenge Weber’s formulation by integrating a material-informatic analysis (see [3] on Bayesian mechanics and constraints on statistical manifolds). Notably, translations of “probability” in Weber range from “ability” to “capacity”, aligning with our possibilistic framing). We introduce novel perspectives on scripts and attention by linking power to the physics of beliefs [3] and the metaphysics of social organization—where metaphysics is understood as the possibilistic exploration of state spaces (i.e., the future).

This article proceeds as follows: First, we briefly outline various philosophical and sociological perspectives on power, focusing on its modulation via scripts—ranging from shared metacognitive conditions to coercive domination. Section 3 introduces AIF as our theoretical foundation. Section 4 synthesizes these insights to analyze social scripts in relation to narratives, attention, coercion, and resistance. Section 5 examines power through the lens of AIF, leading to the formal integration in Section 6. We conclude by discussing attentional strategies that enable alternative modulations of power.

## 2. Linguistic and Behavioral Attractors: The Habits of Shared Attention

(Gravitational) metaphors are prevalent across linguistic expressions and conceptual frameworks [4,5,6,7]. For instance, we frequently describe social interactions in terms of attraction, influence, or “pull”, mirroring physical gravity. These linguistic habits suggest a deeper analytical connection between symbols; semantics—e.g., cultural representations or metaphors—and the constraints of physical phenomena—such as gravitational forces structuring our perceptual interpretation of space. Pierre Bourdieu famously framed power as social *attraction*, transposing the metaphor directly from physical field theory. (As electromagnetic fields structure forces on particles, Bourdieu conceptualized social *fields* as structuring agency via access to economic, cultural, and symbolic capital [8]. These “fields” define power, influence, legitimacy, etc., while what he termed *habitus* would determine their navigation, akin to *satisficing* heuristics formalizable through AIF). Comparably, network science describes preferential attachment processes as common to various phenomena (at the social or individual level) where a scale-invariant analysis can prove useful in describing and explaining the nature of “social and economic disparities governing competitive systems” [9]. Building on this background, we frame power in terms of attentional *attractors* revealing themselves through scripts that guide system configurations, and we analyze these dynamics through AIF.

We can thus reinterpret the dialectic between individual and collective cognition. Information-processing limits at the level of the single agent can be understood as the constraint leading to distributed cognition in sociocultural systems [10], possibly enhancing epistemic confidence through shared interpretation and action [11]. Division of (cognitive) labor thus enhances systemic efficiency, as shown in biological systems [12], robotics [13], and energy resource management [14]. In contrast, it has been proposed that individualized power fosters social coercion by reinforcing self-other divergence [11], and the lack of responsible oversight resulting from downstream power effects at the level of vast social systems can lead to social decoherence [15], resulting in fragmented attention regimes.

A central theme tying these processes to social power dynamics seems to relate to the notion of attention, which we address briefly below and in later sections before turning to AIF in Section 3. Social control begins at the level of attention—by attracting (“follow me!”) or directing it (“look at that!”), whether through explicit commands, or implicitly, through behavioral and habitual cues [16]. Our aim is to formalize aspects of power dynamics by analyzing collective *scripts* and how these modulate attention. Shared structures of meaning can serve as metacognitive computing conditions—e.g., where agents recognize script implications and can collectively deliberate upon them—and/or coercive domination—where scripts subsume agents against their long-term uncertainty-minimization. In the public political realm, we often observe the effects of scripts as “fast and frugal” heuristics [17,18], where scripts organize attention via attractive gestures to a simplified future, or allusions to the past, i.e., scripts which are interpretationally low-cost, cognitively speaking [19]. Sustained cognitive effort is required for complex disambiguation, which is energetically costly [19], and limited organisms need to ensure their integrity remains within bounds that do not exceed free-energy constraints [20]. Given that effective narratives exert significant influence, analyzing power through attention seems adequate, especially when understanding how simplified, low-cost narratives dominate attentional economies by compressing complex realities into low-computation, predictable visions.

The constraints of social power (as and through attention) are therefore both material and informational: whether resulting in territorial or behavioral control, or in the allocation of resources towards (computational) projective anticipations. Social power hinges on controlling scripts (e.g., as specific civic or labor policies, or as narratives, identities, representations, etc.), with computation as its substrate—understood as information processing; as the negotiation of salience in the social realm—both in the flesh and in silico [21]. Section 4 explores how social power, as attentional attraction and (distributed) information processing, operates via scripts that direct attention and enable policy enactment. Many variables modulate attentional regimes, in Section 5 and Section 6, we examine how attentional attraction and synchronization shape social power. Empowerment will therefore be framed as *potential influence* over an environment, quantified via mutual information between actions and states. Power as *potential*—as the ability to do **work** in both physics and socioreproductive contexts—is formalized through AIF where, in our analysis, state space exploration is guided by collective attention to projective possibilities. Formalizing attention mechanics and the complexity of their constraints risks reductionism, but is essential for understanding a possible “new economy of power relations” [22]. In socioconstructive contexts (whether communities, institutions, organizations, states, etc.), agents under more immediate thermodynamic duress resort to shorter-duration policies and/or reliance on dominant narratives promising certainty: agents with infrastructural stability have greater leverage over future possibilities. This presents an important consideration for modeling social power dynamics, where the analysis of existing social asymmetries must always be kept in frame.

We now present the theoretical framework, leading into the formal structuring of scripts, to demonstrate how controlling attention equates to exerting social power.

## 3. Active Inference

AIF posits that biological systems that persist can be described as if they minimize free energy—an upper bound on surprise given sensory data. This principle serves to explain how agents (cells, brains, organisms) actively align internal models with their environments for adaptive behavior. As an enactive process, perception leads to belief updates based on sensory inputs, while action reduces prediction errors by shaping the environment to match expectations [20].

AIF integrates predictive processing and (approximate) Bayesian inference, casting self-organizing systems as hierarchical generative models predicting sensory inputs across spatiotemporal scales. From this 5E (extended, enactive, embodied, embedded, ecological) perspective, beliefs are dynamically formed at multiple scales, with agents optimizing interactions to reduce uncertainty and maintain homeostasis, allostasis, or homeorhesis [21]. Generative models—a core concept in AIF—serve to represent the environment’s structure and guide (social) behavior, integrating perceptual, cognitive, and motor processes.

These models consist of hierarchical representations generating predictions about sensory states based on *inferred* hidden causes. To distinguish between predictions and the data they are modeled from, we speak of generative *processes*, which describe the actual (unobservable) world states that result in sensory observations, while generative *models* encapsulate internal hypotheses about these processes: these are the learned structures that unfold as optimized agential functions. *Learning* emerges as agent actions correct predictive mismatches [23], with message-passing algorithms mimicking neuronal processes [24].

Hierarchical organization enables higher predictive levels to model abstract causes (e.g., intentions), while lower levels track sensory-motor states. Predictions cascade through layers via conditional dependencies, with updates refining beliefs about states. Key components of generative models include sensory states, hidden states, and parameters. Sensory states (*o*) are observed inputs the model predicts, represented as P(o∣s,θ). Hidden states (*s*) are latent variables inferred to explain sensory inputs, minimizing uncertainty, with distribution P(s∣θ). Parameters (θ) are structural dependencies shaping hidden-sensory state relationships, updated over time. The likelihood function P(o∣s,θ) measures how well hidden states and parameters explain data. The prior distribution encodes beliefs before observation, while the posterior updates these beliefs using Bayes’ rule. Model evidence (i.e., marginal likelihood) quantifies the probability of data under the model, aiding model selection.

### 3.1. Free Energy Minimization

Free energy minimization is the unifying principle that motivates perception, cognition, and action in AIF. Parr and Friston’s 2019 paper suggest that this minimization occurs at both local and more extended scales [24]. Free energy minimization at cellular and cognitive levels reflects larger systemic constraints and efficiencies. This process involves reducing the *divergence* between an agent’s predictions and its sensory inputs, maintaining a state of low surprise or entropy.

Free energy is an upper bound on the “surprise” (or negative log-probability) an agent experiences given its observations (*o*). Surprise reflects the degree to which sensory data diverges from an agent’s predictions, but it is generally intractable to compute directly. *Variational free energy* (VFE), a tractable bound on log model evidence applied by Friston and others to the study of the brain [20,25,26], recasts this intractability as an optimization problem by using an internal generative model, represented as *q*-distributions over hidden (latent) states (*s*), to approximate the posterior distribution. The variational free energy *F* can be expressed as

(1) $$\begin{array}{cc} F & =\underset{\mathrm{Divergence}}{\underbrace{D_{\mathrm{KL}}\left[q\left(s\right)\;∥\;p\left(s|o\right)\right]}}-\underset{\mathrm{Surprise}}{\underbrace{\ln p(o)}} \end{array}$$

(2) $$\begin{array}{cc} \; & =\underset{\mathrm{Complexity}}{\underbrace{D_{\mathrm{KL}}\left[q\left(s\right)\;∥\;p\left(s\right)\right]}}-\underset{\mathrm{Accuracy}}{\underbrace{E_{q(s)}\left[\ln p\left(o|s\right)\right]}}. \end{array}$$

Here, $D_{\mathrm{KL}}\left[q\left(s\right)\;∥\;p\left(s|o\right)\right]$ is the Kullback–Leibler (KL) divergence between the approximate posterior q(s) and the true posterior p(s∣o). Minimizing this term aligns the agent’s beliefs (encoded in *q*) with the posterior distribution. The −lnp(o) term represents the surprise or the negative log model evidence of an observation *o*.

$E_{q(s)}\left[\ln p\left(o|s\right)\right]$ represents the expected log-likelihood of observations given the hidden states. Maximizing this term makes the agent’s model predictive of actual sensory inputs. To minimize free energy and align their sensory inputs with their predictions, agents have to update their beliefs about the world. Belief updating under AIF is achieved through variational Bayesian inference, whereby agents continuously update their beliefs about the state of the world based on new sensory information. This allows agents to make predictions and adjust their actions to confirm these predictions, thereby preserving, e.g., a coherent sense of *self* in a changing environment.

Given adequate belief updating (which is context- and observer-dependent), the system can then select actions that are consistent with its internal generative model. This effectively ensures that the chosen policies minimize future free energy. This mechanism has broad implications, enabling goal-directed behavior and coherent action selection, even in novel environments. Minimization occurs through two complementary strategies: *perceptual* and *active* inference, which are inextricably linked. Perceptual inference updates beliefs about hidden states to align with sensory data, akin to online learning. Active inference extends inference to the environment, seeking to actualize beliefs, modifying the environment, or seeking new data to better align sensory inputs with internal models, and this creates a feedback loop where actions result in the reduction of prediction errors. Distinguishing between internally driven and externally imposed processes poses challenges, especially if we consider how, in complex social coordination, individual actions are guided by internalized logics acquired through already established habits. Free energy minimization, as a grounding principle, may help reasoning about this in its capacity to reflect systemic efficiencies through biological scaling laws, optimizing internal states with minimal energetic cost [10]. Crucially, complex adaptive systems cannot remain optimized on their long-term average performance without occasional short-term disruptions or investments [27,28,29]. Long-term stability, resilience, or growth is, in fact, an emergent property sustained by intermittent episodes of instability or intense effort [30,31]. For example, biological evolution depends on mutations and selection bursts to climb to higher fitness peaks [32,33]; brains optimize computational efficiencies by teetering on the edge of instability, allowing bursts of neuronal activity [23,34]; ecosystems remain productive by experiencing disturbances that recycle resources [30,31]. In each case, short-term high-risk or high-energy actions can act as “rejuvenating fires” or exploration probes, preventing the system from settling into suboptimal equilibria or, ultimately, decaying and dissipating [27,35]. At various scales, this principle manifests differently. Cells maintain homeostasis by modulating biochemical pathways in response to deviations from expected states. Cognition employs predictive coding, where sensory inputs are compared against hierarchical generative model predictions, with mismatches (prediction errors) triggering updates. Free energy minimization effectively reduces (relative) entropy, aligning beliefs (internal models) with reality (sensory inputs).

Complex agents can also predict their own actions, forming plans optimized to achieve desirable outcomes. This shifts from minimizing VFE to EFE, which decomposes into extrinsic value (goal-directed rewards) and epistemic value (uncertainty reduction). Minimizing EFE balances exploration (gathering information) and exploitation (goal-directed action), optimizing adaptive behavior across time. It is expressed as

(3) $$\begin{array}{c} G=-\underset{\mathrm{Epistemic}\;\mathrm{value}}{\underbrace{E_{q(s,o|\pi )}\left[D_{\mathrm{KL}}\left[q\left(s|o,\pi \right)∥q\left(s|\pi \right)\right]\right]}}-\underset{\mathrm{Pragmatic}\;\mathrm{value}}{\underbrace{E_{q(o|\pi )}\left[\ln\tilde{p}\left(o\right)\right]}}, \end{array}$$

where $\ln\tilde{p}\left(o\right)$ represents the agent’s preferences over observations or *extrinsic value*, expressed as a (biased) prior probability distribution over observations [36]. The first term represents the (negative) *expected information gain* or *epistemic value*, which scores the degree to which an agent expects to update their beliefs in light of new sensory data. By pursuing outcomes that maximize a balance between epistemic and extrinsic values, agents simultaneously satisfy immediate goals and resolve uncertainty, driving adaptive behavior in complex environments.

### 3.2. Why Active Inference Is Suitable for Modeling Power Dynamics Through Attention

AIF seems well-suited to understand power dynamics, beginning with individual agents as attentional, predictive systems that minimize uncertainty through perception and action [37]. These agents develop control through embodied interaction with their environment, establishing what Bruineberg calls the attentional “primacy of the ‘I Can’”—the fundamental ability, or *power*, to influence one’s surroundings. This attentional control scales up through generative models that enable agents to predict and understand others’ intentions [10,38], where attention is fundamentally shaped by deep preferences and established social habits that determine what becomes salient in the environment [39].

At the social level, we can consider how power dynamics emerge through shared predictive models and environmental mediation. Groups coordinate via shared narratives [40], while stigmergic processes, decentralized mechanisms of indirect coordination, shape collective behavior through environmental modifications [41]. Deontic cues, cues dealing with normative statements about what ought or ought not to be done, seem to reinforce social norms [42], and established cultural practices shape attention, creating shared perceptual landscapes [10,43]. These processes can enable efficient coordination through joint attention and mutual understanding of environmental affordances.

Social groups tend to self-organize based on belief similarity [10,42,44], and communication network effects have the capacity to amplify or marginalize coordinating scripts [45]. However, the assumption of social *sameness* in cultural niche-formation—an assumption which structures dominant narratives—crucially becomes a political question, i.e., a question of power and social organization. If attention is shaped by deep, embodied preferences and by shared scripts (narratives, identities, histories), both of which determine environmental salience, then the ensuing power dynamics can be critically examined through formal methods, in an effort to prevent homogenization and ensure evolving, diversifying possibilities in cultural landscapes. Social structures such as institutions, for example, promote script legibility by directing attention toward specific narratives [46], and this can be understood as an effort through which a coordinating balance is explored: a balance between the coordination of benefits (for the institution and its members) and the management of diversity (which might lead to members distancing themselves and therefore weakening the institution). In the dynamics of institutional narratives (such as those of the state), phrases such as “There is no alternative”, or at the communicative social level, multiply-interpretable anaphora, which are presented as having a single, true meaning, can be understood to exemplify one-dimensional visions that oversimplify complex systems, often to their detriment [47]. This is because they can result in the suppression of the (cognitive) diversity that can be understood as that which in fact maintains a social system in adaptive, functioning order, as exemplified for various other evolutionary systems described earlier. Studies have shown that sufficient (cognitive) diversity seems to enhance effective collective intelligence by endowing groups with multiple perspectives in problem-solving scenarios, while too much diversity can lead to group decoherence [48] (hence the sometimes observed institutional rigidity that leads to social dynamics oversimplification). Power, when framed in terms of *responsibility*, can be understood to promote more prosocial effects [15], though in the context vast regimes of attention, expecting structural institutional power to be capable of accounting for the diversity of perspectives that ought to be regulated by it remains a paradoxical issue: an institution is precisely a social device that ought to simplify a complex whole into a coherent system, hence its capacity to diminish diversity.

Considering the complex dynamics of multiple agent estimations coordinating attention towards the production of the future, social power can be reframed as *possibilistic*, making bets under high-uncertainty and thus amenable to treatment under our framework. Social power is a distributed process leading to the emergence of attentional dynamics that influence the possibility for members to direct their attention in specific ways, and/or to perform actions that strengthen or weaken established social powers. Given life’s hierarchical organization—both physically and conceptually—understanding distributed generative models over time (e.g., generations, historical narratives) offers a framework beyond individualized voluntarist freedom and unit or atom-based agency, focusing instead on collective dynamics and their (probabilistic) mechanics. Social power can thus be formalized as a matter of the collective coordination of attention [49], and attending to this through AIF reveals novel modeling paradigms to analyze its complexities in further detail.

## 4. Social Scripts and Power

### 4.1. Scripts as Frameworks for Social Behavior

Social scripts have been conceptualized as the blueprints for navigating social situations, guiding behavior through internalized cognitive schemas and externalized social structures [49]. They encode socially shared knowledge, variants of expected *sameness*, prescribing behavioral standards, akin to a dramaturgical script. Strong scripts specify structured behavioral sequences with causal and temporal order (e.g., the North American restaurant script: seating, ordering, eating, paying) [49]. Weak scripts outline general features of events without rigid sequencing, enhancing situational interpretation and action selection through semantic associations. Scripts can reinforce power structures by normalizing behaviors and making deviations seem abnormal [42]. They can establish predictable social interactions, often benefiting those already in power by encoding and reproducing (hierarchical) roles [50]. Of course, note that, in general, scripts can benefit social interaction as a whole by aiding effective communication and reducing the potential for conflict. When and how agents actively (e.g., reflexively, i.e., consciously or critically, or collectively, deliberately) engage with norms or simply perform them remains an open question.

Under AIF, scripts can be understood to function as the attractors of shared generative models, allowing predictive coordination in social behavior. They encode possible causal structures, reducing uncertainty about the complexities of social interactions. Deontic cues embedded in scripts guide action selection by making certain policies more likely in specific contexts [42]. Scripts transmit across generations via cultural learning, shaping the reproduction of social structures through both explicit instruction and implicit environmental adaptation.

### 4.2. Narratives as Scripts

(Semantic, historical, ideological, etc., i.e., *cultural*) narratives may, again, function as scripts that normalize behavior by providing templates for “appropriate” action, shaping social meaning and justifying existing power structures [42]. Under any narrative, deviant behavior may be curiously engaged, because it is seen as valuable and explorative (e.g., most art), or it may be considered inappropriate (e.g., graffiti), leading to castigation or neglect. Again, here the key question remains whether agents critically engage with norms or merely perform them. Given that engagement with or performance of them is highly context-dependent (it can vary depending on sociocultural circumstances, cognitive state, etc.), formalizing attention in terms of that which leads to power-pooling has the benefit that it can simplify these intractable landscapes towards novel understandings (albeit reductively and thus inevitably). In AIF, narratives function as cultural affordances, predictions that simultaneously model and therefore shape future states, i.e., potential configurations of reality that have not yet occurred but are anticipated or expected (in greater or lesser detail). Future states can encompass potential technological developments, market behaviors, and social adoption patterns. Narratives, which connect agents through attention mechanisms, reduce uncertainty in two ways [51]. First, by providing priors that constrain the hypothesis space of possible futures (creating a sense of collective knowing). Second, by actively influencing events through behavior modification. The latter process is how narratives modulate the landscape of predicted precision by making certain actions appear more valuable for uncertainty-minimization. In consequence, agents preferentially sample evidence that confirms the existing, dominant narrative-based predictions, reinforcing loops where coordinated behavior increases the probability of narrative-consistent expectations. Narratives, therefore, go beyond “mere” representational effect, but are instrumental in shaping the outcomes of social-attentional dynamics.

The effects of free energy minimization at more fundamental linguistic scales can be understood to manifest through semantic attractors like redundancy, rhyme, etc., which aid memory, communication, and cognitive efficiency by establishing structures of reiteration. Rhythmic structures in speech facilitate information storage and retrieval, reducing uncertainty about the coordination of semantic spaces. This effect, driven by the limits of energy-efficiency, reveals how, at social scales, dynamics can normalize certain behaviors by favoring easily predictable, ‘digestible’ narratives. Unlike shorter-range online predictions [52], where errors present immediate challenges, the offline exploration of narratives represents collective speculative spaces for model exploration without “real-world” risks. In the context of complex communication and social coordination, problems arise when dominant narratives prioritize, e.g., mere power acquisition in favor of self-preservation, which has been examined as leading to misinformation, echo chambers, and policy justifications based on assumed conditions [45]. This is particularly problematic when the promotion of compelling narratives is enforced by those who are *not* suffering the consequences of said narratives: those under higher thermodynamic duress (socioeconomic, corporeal, etc.). In general, there is a clear tendency for simpler narratives to prevail due to the cognitive, corporeal, etc., constraints that limit complex exploration.

In this way, narratives may shape belief-updating landscapes, influencing world-state exploration. Since humans naturally share narratives, this process is integral to AIF in social systems. This framework has significant implications for narrative cognition and collective behavior scripting. We now turn to an analysis of power through AIF.

## 5. Power Through the Lens of Active Inference

### 5.1. Individual Agency and Empowerment

Autonomy refers to a system’s capacity to govern itself based on internally defined norms [53]. Autonomous behavior emerges through entropy maximization under constraints [54]. Agents do not simply maximize randomness; agents optimize their behavior by maintaining a balance between maximizing entropy while bound to the limits of respecting systemic constraints [55]. This is consistent with Jaynes’ principle of maximum entropy, which states that, among all probability distributions that satisfy the known constraints (such as data, moments, or other conditions), the least biased estimate is the one that maximizes entropy.

In autonomous agents, these constraints include both the agent’s internal model and environmental dynamics [56]. We can think of *empowerment* as a formal measure of an agent’s control over its future states, defined as the maximum mutual information between an agent’s actions and the resulting states [57]:

(4) $$\begin{array}{c} E_{t,n}=\max_{p(a_{t,n})}I\left(A_{t,n};s_{t+n}\right), \end{array}$$

where $I(A_{t,n};s_{t+n})$ denotes the mutual information between the next *n* actions $A_{t,n}$ from time *t* and the state $s_{t+n}$ at time t+n, and $p(a_{t,n})$ represents the probability distribution over the next *n* actions from time *t* [57]. In this context, agents select actions that maximize their control over future environmental states, optimizing their ability to influence outcomes. Higher empowerment indicates greater potential for an agent to achieve desired future states. Importantly, empowerment can be decomposed into entropy terms as

(5) $$\begin{array}{c} I\left(A_{t,n};s_{t+n}\right)=H\left(A_{t,n}\right)+H\left(s_{t+n}\right)-H\left(A_{t,n},s_{t+n}\right), \end{array}$$

where H[X] represents the Shannon entropy of the random variable *X* and H[X,Y] denotes the joint entropy of *X* and *Y*. This decomposition reveals that empowerment involves maximizing the marginal entropies of state and action distributions while maintaining strong correlations between actions and their consequences [54]. The joint entropy represents the total uncertainty in the pair of actions and future states. If actions strongly constrain future states, the joint entropy approaches the entropy of the action sequence, which in turn increases the mutual information. (We would like to thank Alex Kiefer for pointing out this breakdown of empowerment in terms of entropy, and for suggesting the broader idea of casting intrinsic motivation as constrained entropy maximization). We can also link these ideas to recent developments in understanding intrinsic motivation in dynamical control systems [58] and complex behavior emerging from the drive to occupy future action-state path spaces [59].

Empowerment as defined above is *intrinsic* motivation that does not specify how an agent should take actions to achieve specific goals. Extensions have been introduced to incorporate goal-directedness by introducing a reward constraint [60] or tradeoff [61], which highlight the tensions that may arise between intrinsic and extrinsic motivators.

Under the AIF framework, autonomous agents select policies (sequences of actions) by minimizing expected free energy (EFE), which includes both epistemic (information-seeking) and pragmatic (goal-directed) terms [36,62]. Active inference differs from empowerment in several respects, but one crucial point of difference is that it explicitly models the partial observability inherent in many decision-making environments, motivating agents to resolve uncertainty through curiosity-driven behavior. This balance between exploration and exploitation allows agents to both gather information about their environment and pursue specific goals. Policy selection in autonomous agents involves maintaining a generative model of environmental dynamics. To do so, agents must update their beliefs about current states through perceptual inference. They can then select actions that minimize expected free energy over future states and ultimately learn from action outcomes to improve the generative model. Both empowerment and EFE highlight different components of power that are relevant to the present discussion. Empowerment more directly formalizes the notion of an agent’s control over its environment, but typically considers fully observable environments, thus ignoring considerations of epistemic foraging to reduce uncertainty about the state of the environment. On the other hand, the EFE emphasizes the role of information gain in the context of generative models, pointing to the importance of understanding how informational landscapes and affordances can be shaped by those who wield social power. However, this formalism less directly captures the notion of influence that an agent has to shape its environment, which is desirable for a formal definition of power. Developing a unified framework for integrating these different objectives can provide a deeper understanding of how power, goals, and information relate to one another.

### 5.2. Social Power Dynamics

Under this view, social power dynamics emerge as a function of interacting perception–action loops across multiple agents. Power is the ability to shape both the physical and informational environment. Power operates as an attractor in social systems, creating a force that draws agents toward centers of influence. This gravitational effect works through two primary mechanisms. Entities with high power gravity naturally draw others to them through access to resources, information, or strategic advantages. As more agents are drawn into their “orbit”, their influence amplifies through positive feedback loops, e.g., through phenomena such as preferential attachment.

Powerful entities may offer perceived stability and predictability in an uncertain environment. In AIF terms, they help other agents minimize their expected free energy by providing frameworks for prediction, essentially by dictating the information geometry landscape [42]. Power might thus confer significant advantages in information processing and management, as powerful entities occupy positions that grant them privileged access to information streams, potentially enabling more accurate modeling of their environment. Ultimately, they also control what can and cannot be modeled, and in what way, through their position and resources. Powerful agents can offload computational demands onto others or their environment, allowing for more efficient processing of complex information. In the globalizing condition unfolding over the 20th century, we can understand these dynamics as the emergence of novelty- and complexity-generating networks based on agent-interactions—among these, e.g., the financial domain or the information-attention economy—which can often be observed as continuing to provide benefits to those with leverage over its structures. It is primarily agents already well-positioned within the informatic domain, who have leverage over attention regimes. (As observed by [15], both distance from subordinates and therefore regimes of influence, as well as competitiveness, can lead to detrimental antisocial power effects (see Hypothesis 4 in the cited paper)). Power influences how uncertainty is processed within social systems: powerful entities can better reframe estimations due to their broader resource base and influence over narrative construction. Through their ability to shape both physical and informational environments, powerful agents can actively reduce uncertainty for themselves while potentially (and often inevitably) increasing it for others. Though some literature on the prosocial, or socially cohesive, possibilities of power exists, observing power as constrained by the landscape of social norms and moral obligations, which have the capacity to incline powerholders to “act benevolently toward others” [15] and induce a sense of responsibility that could alleviate power’s asymmetrical top-down control effects, is a view that remains understudied, “and the precise mechanisms by which this prosocial effect is brought about remain unclear” [15]. Because modern power operates at the global level, where many of its downstream effects are uncontrollable by powerholders, we therefore frame power as inevitably coercive, but suggest a few ways in which the phenomenon can be deconstructed in order to better understand how it could be modulated towards more prosocial effects (as suggested by [15], *attention* is a key factor here).

The manipulation of information geometry—both in terms of perception and action—we believe is central to how power operates in social systems. Powerful entities may have the ability to shape the attentional landscapes of others, directing focus and resources toward certain states while making others less salient or accessible. This ability to manipulate both the physical and informational structure of the environment creates a self-reinforcing cycle where power begets more power through increasingly sophisticated control over the collective attentional landscape. Power also expands an entity’s available state-space policies in several ways: powerful entities have access to a broader range of possible actions and strategies through their resources and influence. Through their attractive force, they gain access to the state-space policies of agents within their sphere of influence, effectively expanding their operational capabilities.

### 5.3. Formal Framework Integration

Power dynamics within and between (groups of) agents can be formalized by addressing various dimensions of control, influence, and synchronization. At the individual level, recall that *empowerment* quantifies an agent’s possibilistic influence over its environment by assessing the mutual information between the agent’s actions and the resulting states [57].

In active inference, policy selection is driven by the EFE, which focuses on specific epistemic and pragmatic motivations to achieve one’s goals and resolve uncertainty about one’s environment. This more closely resembles the notion of goal-directed empowerment [60], which adds a minimal performance constraint to the empowerment objective.

In social contexts, active inference models account for the influence of social norms and expectations via *deontic value* [42]. This value, derived from specific *deontic cues*, modulates policy selection alongside the pragmatic and epistemic considerations afforded by the expected free energy *G*. This deontic value is formalized as the posterior probability of selecting a policy π given a deontic cue *o*:

(6) $$\begin{array}{c} P(\pi |o)\propto \exp(-G(\pi )+\log P(o|\pi ), \end{array}$$

where G(π) represents expected free energy for a policy π, and logP(o∣π) is a likelihood capturing how well the hypothesis “π is the appropriate/expected policy” explains or fits the observed cue. Social norms modulate policy selection, encouraging behavior that is goal-oriented and aligned with collective expectations. Deontic value may also be linked to a particular kind of empowerment. Consider that to a given agent, other agents’ actions and policies may be viewed as part of the external state that is being predicted and controlled. Therefore, the empowerment of the agent includes their influence on others’ policies, often exerted via deontic cues, which may be reflected by the deontic value term in Equation (6). If conforming to norms makes an agent’s behavior more predictable, this could increase the ability of other agents to model and interact effectively with them. This potential connection between individual conformity and the empowerment of interaction partners is an interesting avenue for exploration. A deeper investigation into how the two are precisely related is beyond the scope of this paper, but would shed light on the relationship between social norms and individual/collective empowerment.

Beyond control over other agents’ policies, agents may also exert influence over others’ beliefs, constituting another aspect of empowerment that focuses on viewing other agents’ generative models as states external to oneself. In particular, one phenomenon worth investigating is the synchronization of beliefs between agents in social interactions. This can be quantitatively assessed through the *Kullback–Leibler divergence* between different agents’ generative models. In the simple case of two agents *i* and *j*, this is simply

(7) $$\begin{array}{c} D_{\mathrm{KL}}\left[q_{i}\left(s\right)∥q_{j}\left(s\right)\right]=E_{q_{i}\left(s\right)}\left[\ln q_{i}\left(s\right)-\ln q_{j}\left(s\right)\right], \end{array}$$

where $q_{i}\left(s\right)$ and $q_{j}\left(s\right)$ denote the belief distributions of agents *i* and *j* over the hidden state, respectively. This quantity reflects the degree of belief alignment, indicating the extent to which a *shared* understanding emerges from social interaction. The ways in which this is unconscious, tacit, or otherwise analytically unattainable are the elements that lead to many of the intractable aspects of formalizing these dynamics. (This is a consideration which ought to be explicitly stated in any modeling attempt).

Belief alignment itself can emerge from reciprocal interactions governed by the minimization of variational or expected free energy. In social exchanges, as agents update their beliefs in response to outcomes generated by each other, it may be the case that parts of their generative models become synchronized, establishing a shared narrative or even a sense of sameness. This may arise as agents select actions consistent with their internal models, and thus produce outcomes that align with their beliefs, which reciprocally influences the beliefs of their counterparts. Over successive interactions, the minimization of *expected* free energy can lead to stronger synchronized beliefs (encapsulated by the KL divergence), by a process of mutually adapting beliefs and behaviors in order to achieve benefits from coordination, which includes both pragmatic and epistemic components (In adversarial or mixed-motive settings, this synchronization need not occur, e.g., in situations where an agent is being deceived or manipulated by another). Repeated exchanges may effectively reduce this divergence:

(8) $$\begin{array}{c} \lim_{t\to \infty }D_{\mathrm{KL}}\left[q_{i}^{t}\left(s\right)∥q_{j}^{t}\left(s\right)\right]\approx 0, \end{array}$$

where the superscript indicates the generative model of each agent at different points in time. Intuitively, under appropriate interaction conditions, agents share beliefs (typically in the form of likelihoods) with one another and incorporate these into their own generative models, thus bringing their states of belief into alignment with each other. This signifies a stable convergence where the beliefs of both agents synchronize, establishing a shared understanding and a common ground for interaction. There are many ways this synchronization can occur, with extreme cases being where

(9) $$\begin{array}{c} \lim_{t\to \infty }D_{\mathrm{KL}}\left[q_{i}^{t}∥q_{i}^{0}\right]\ll \lim_{t\to \infty }D_{\mathrm{KL}}\left[q_{j}^{t}∥q_{j}^{0}\right], \end{array}$$

or vice versa. Intuitively, these correspond to situations where one agent makes a small update to their beliefs but causes the other to change their beliefs to a greater extent to fit their own. Leaning on the gravitational metaphor for our account, the agent who makes the significantly smaller update may do so as a result of being in a position of greater social power.

Linking this idea back to *empowerment*, notice that defining “external states” is more complicated in social contexts, because one agent’s internal state is another agent’s external state, and vice versa. This means that different agents’ internal representations of the world must be similar in some parts (those that are external to all of them) and different in other parts. Therefore, part of what it means for an agent to maximize their empowerment in a social context is to actively make parts of other agents more predictable and useful to themselves, which is often more easily achieved by bringing other agents’ generative models in line with one’s own (through attentional scripts).

In general, belief alignment can emerge as a natural consequence of each agent’s drive to minimize free energy attuning to a social context. This fosters synchronized behavior, shared narratives, and collective understanding, unifying agency, social influence, and collective behavior under the cohesive formalism of AIF [38,63]. However, as the brief analysis above reveals, such synchronization through belief alignment may come about as a result of the exertion of social power, highlighting the importance of understanding not just when this synchronization occurs, but also how it does so.

As we conceptualized it, power is an agent’s capacity to shape and control the (semantic) attentional “attractors” within a social-informational landscape. These attractors represent stable states or patterns that agents are drawn towards (scripts, narratives, representations, etc.), which reduce uncertainty and create predictable social structures. Power enables agents to influence which states become salient or preferred for others, thereby controlling the flow of attention, shaping beliefs, and modulating the precision of predictions and therefore the production of future collective states. The rise of marketing, as a field—during the 20th and increasingly fueled by digital technologies in 21st century—has shown, time and time again, especially in the last 20 years, how mediatic domination (narrative control) results in the shaping of attractors that organize life. Those with leverage on attentional scripts, whether it is political narratives broadly cast or subliminal marketing on social media, influence the behavior of those attending to them.

An agent with power has the capacity to shape these expected outcomes by adjusting the social or informational environment in a way that amplifies certain states while suppressing others. For example, powerful agents may create narratives, i.e., scripts, that reduce the dimensionality of the state space by establishing strong, predictable attractors—such as social norms or shared narratives. By doing so, they effectively direct attention and ensure that other agents align their actions with these preferred states. Reinforcing a pattern where the world is modeled according to their internal logic, and imparting this logic on the generative models of others, makes them ever more predictable and therefore manipulable.

We can formalize this influence on decision-making by noticing that, according to the decomposition of the expected free energy into pragmatic and epistemic components, the ability to shape narratives so that people believe that their goals are being achieved and uncertainty is being resolved can influence what policies they end up enacting. Beyond this, influence by agents on the perceived reliability of different pieces of evidence can amplify the reach and impact of certain beliefs and actions across a social network.

The ability to direct attention through attractors and precision modulation defines power in AIF, as these mechanisms can be used to shape the beliefs and preferences of agents. Recall that one simple way of formalizing this process is by analyzing the KL divergence between agents’ belief distributions. A lower KL divergence value indicates a closer alignment of beliefs. In leader–follower dynamics, it has been found empirically that a leader minimizes this divergence by exerting control over precision, amplifying others’ confidence in specific interpretations of their sensory data. For example, if the leader’s goal is clearly defined and they select social epistemic actions (more informative, albeit less efficient actions), they convey their intentions early and reduce uncertainty for followers [52]. This approach encourages other agents to update their beliefs to align with the leader’s, effectively lowering KL divergence across the group. Even in cases where leadership is not predefined, an agent can assume a leadership role by adjusting precision to show high confidence in their beliefs or goals [52]. This increase in precision draws other agents toward the confident agent’s stance, resulting in a convergence on shared beliefs. Precision-driven leadership can thus promote belief alignment without coercion, as agents gradually follow those who express strong, reliable predictions, or as those wielding power responsibly attend to the effects of their actions [15]. In this way, the power to direct attention and structure narratives, and therefore modulate precision, enables agents to actively shape collective beliefs, creating cohesion within groups and guiding group behavior through the minimization of belief divergence.

The applications of this inevitably begin at reductive formalizations (and the math is never the territory [64]); however, the implications of these arguments for complex social dynamics in the realms of, e.g., policy and governance are vast. Coordination dynamics at the level of simple perception–action cycles, such as the ones analyzed in [52], need to be framed in terms of their possibilities for novel understandings of social change processes. If we are social agents that benefit from collective coordination, how can an analysis of these dynamics through attention mechanisms help us understand the nature of this coordination (of power)?

The formalisms of empowerment and AIF can be applied to real social phenomena by providing a set of data structures and algorithms that can be used to predict and understand features of social interactions. For example, in an organizational scenario, suppose that each state is a distribution of tasks assigned to different agents, and let actions be decisions or requests made by the leader. By measuring the empowerment of the leader, the organization is better able to understand how strongly the leader’s decisions influence the outcomes that actually materialize. Conversely, one could measure the empowerment of agents in an organization to understand the degree to which they have bargaining power with the organization. Under AIF, the variational free energy and expected free energy equations provide a mathematical account of the mechanics of belief and policy updating processes. These are useful in building simulations to predict the effects of different social interventions on social power dynamics. Belief alignment processes may be measured by, e.g., running repeated polls or tracking repeated opinions within a population. Studying the dynamics of such beliefs and how they relate to the stances of socially powerful entities may be helpful in developing better models of the mechanics of social attention.

Another starting point for the extension of these formalisms to multi-agent interactions is using the language of game theory. In particular, the concept of Free-Energy Equilibria (FEE) captures the notion of a joint policy in which all agents in a given interaction are minimizing their free energy [65]. Under certain conditions, it may be expected that interactions between locally free-energy minimizing agents tend to drive the system toward FEE. However, as is well understood in game theory, there are typically many equilibria of a game, and depending on the starting point and interaction/learning dynamics, the system may converge to undesirable equilibria where a handful of agents exert significant control over others to their detriment, due to possessing significant degrees of social power. An important area of future study is thus to first establish a better understanding of what makes different equilibria more or less societally desirable, and then to understand how incentive landscapes may be shaped to navigate society toward better equilibria. Shared narratives, where agents are in the know about what is shared, are fundamental here, and this begins by paying attention to the (possible) rules in place, hence the need for formal methods. This is part of what critical metacognition entails: knowing how we are collectively navigating uncertainty, and taking account of already-given asymmetries. In the conclusion below, we explore a few salient implications of our arguments.

## 6. Conclusions

Attentional power dynamics shape the probability landscape of future possibilities. In this article, we have aimed to provide conceptual clarity around social power, and identified mathematical frameworks in which to begin formalizing and measuring different aspects of social attention. Our proposal to address possibilistic power, grounded in AIF, offers ways to identify, evaluate, and better understand social power distributions as resulting from attention mechanisms. These formalizations can have a significant impact for understanding power dynamics in an increasingly information-driven world. The transition from material to informational forms of power presents plenty of questions and opportunities for possible framings of future social dynamics. This paper examines how AIF can shed light on these shifts, suggesting that (possibilistic) power operates through mechanisms of social association, through the identification of and attention-pooling towards influential agents, as well as through enhanced computing-projective capabilities. Both these phenomena create self-reinforcing cycles, where empowered agents can expand their state space (and, often, minimize cost and future vulnerability). What has been previously framed in terms of territorial/material and cultural power can now also be understood in terms of informational and computational power. Both of these have received attention as forms of capital, a term we can therefore reframe as possibilistic power. This new understanding links the cognitive foundations of narrative engagement and the ensuing social scripts that make or break power relations.

The ontoepistemic challenges in grounding (deontic) value can be understood under AIF in ways that open up complex semantics and social coordination towards a possible naturalization of these phenomena—not in an effort to reduce them to simplistic images, but rather in an effort to provide new entries into as well as possible accountability metrics of thorny ethical landscapes. In our proposal, coordinated behaviors emerge as shared models that reduce collective uncertainty (again, with the caveat that whatever is supposed to be “shared” is not always given nor understood). When scripts become internalized by multiple members of a community, this allows for the collective construction of future outcomes. Following those who appear to have the potential to reduce our uncertainty leads to agents associating with agents and actions that would seem to meet those expectations. Metacognitive criticality, understood as the ability to know and possibly modulate the rules one is subject to, as well as being able to imagine new rules, is an important aspect of how we collectively navigate uncertainty, assuming already given asymmetries. Future research could focus on developing metrics for measuring aspects of possibilistic power, and perhaps evaluating how attentional interventions could render novel distribution dynamics of both material and informational resources (which can be treated as highly interrelated, if not as operating under the same constraints [62].

This shift from territorial/material to informational/computational power requires special attention in futurological contexts. We propose that AIF provides crucial theoretical tools for making power structures visible and therefore analyzable, allowing for the potential to rethink current social asymmetries. Given the apparent transition between the organization of life around the centrality of physical work and matter, towards a centrality of attention and information, a sustainable social future requires addressing both resource and information distributions. The latter is, we propose, amenable to analysis through the power of attention as a major factor influencing social dynamics.

Hierarchies are inevitable in complex thermodynamic systems. We propose fostering transparent, plastic hierarchies that remain contestable and therefore open to novelty. This approach acknowledges historic and thermodynamic constraints, while recognizing the importance of cultivating space for systemic evolution. While hierarchies are inevitable, social scripts should be read-writable by those with an interest in survival through them. This helps reorient our understanding of an unfolding, globalizing, interconnected, and interdependent life: participation in a system is only participatory if those involved are able to track the consequences of the variables and thus future state spaces at stake. The collective distribution of information-processing (and, as we have argued, *material-processing*) capabilities is essential for systemic sustainability in complex sociocultural systems. It is fundamental that these dynamics are understood as evolving processes, and not natural givens from which “oughts” are to be derived [66].

## Data Availability

The original contributions presented in this study are included in the article. Further inquiries can be directed to the corresponding author.
